# The influence of a change in the meniscus cross-sectional shape on the medio-lateral translation of the knee joint and meniscal extrusion

**DOI:** 10.1371/journal.pone.0193020

**Published:** 2018-02-15

**Authors:** Piotr Luczkiewicz, Karol Daszkiewicz, Wojciech Witkowski, Jacek Chróścielewski, Tomasz Ferenc, Boguslaw Baczkowski

**Affiliations:** 1 II Clinic of Orthopaedics and Kinetic Organ Traumatology, Medical University of Gdansk, Gdansk, Poland; 2 Department of Mechanics of Materials, Faculty of Civil and Environmental Engineering, Gdansk University of Technology, Gdansk, Poland; Rush University Medical Center, UNITED STATES

## Abstract

**Objective:**

The purpose of this study was to evaluate the influence of a change in the meniscus cross sectional shape on its position and on the biomechanics of a knee joint.

**Methods:**

One main finite element model of a left knee joint was created on the basis of MRI images. The model consisted of bones, articular cartilages, menisci and ligaments. Eight variants of this model with an increased or decreased meniscus height were then prepared. Nonlinear static analyses with a fixed flexion/extension movement for a compressive load of 1000 N were performed. The additional analyses for those models with a constrained medio-lateral relative bone translation allowed for an evaluation of the influence of this translation on a meniscus external shift.

**Results:**

It was observed that a decrease in the meniscus height caused a decrease in the contact area, together with a decrease in the contact force between the flattened meniscus and the cartilage. For the models with an increased meniscus height, a maximal value of force acting on the meniscus in a medio-lateral direction was obtained. The results have shown that the meniscus external shift was approximately proportional to the meniscus slope angle, but that relationship was modified by a medio-lateral relative bone translation. It was found that the translation of the femur relative to the tibia may be dependent on the geometry of the menisci.

**Conclusions:**

The results have suggested that a change in the meniscus geometry in the cross sectional plane can considerably affect not only the meniscal external shift, but also the medio-lateral translation of the knee joint as well as the congruency of the knee joint.

## Introduction

Menisci are crescent-shaped fibrocartilaginous structures that are located between the femoral condyles and the tibial plateau. They increase the congruence between the articular surfaces and they transmit a load across the incongruous tibiofemoral joint, thereby decreasing the mechanical load on the articular cartilage [1,2]. Recent years have seen significant advances in our understanding of the role of meniscus geometry and the meniscus positioning for knee functions [3,4,5]. Many authors have reported that a meniscal external displacement (extrusion) reduces the mechanical protection of the knee cartilage. This causes cartilage loss, knee pain, synovitis and it represents a strong risk factor for the onset and the progression of osteoarthritis of the knee [6,7,8,9]. Meniscus extrusion is a well-known fact in the natural history of knee osteoarthritis [10]. The well documented factors that have been reported as contributing to a meniscal displacement are: a tearing of the circumferential collagen fibres within the meniscal tissue and cartilage degeneration [10,11,12]. However, a degenerative meniscal extrusion is very often connected with a change in its shape, without a disruption of the meniscus [13,14]. It remains unclear why menisci, without any lesions, alter their position and how a change in their shape influences this process [10,13,15].

In a previous work by the authors of this paper [16], analyses of the stress distribution and the menisci deformation were performed on an Open Knee Model [17], with the geometry of the lateral meniscus showing a diminished height. By analysing the resultant contact forces, it was shown that a lateral meniscus had a far less tendency to extrude. The main limitation of the above work was that the assessment of the effects of meniscus height reduction were only conducted in the lateral compartment. The differences in joint geometry between the medial and lateral compartments led to the hypothesis that a change in the geometry of the medial meniscus might not be affecting extrusion in the same way as a change in the geometry of the lateral meniscus. Therefore, a decision was made to additionally analyse the effects of a change in the height of the meniscus on its extrusion in the medial compartment. The Open Knee model that was used in analyses of the first paper was based on Magnetic Resonance Imaging (MRI) scans of a knee taken from a 70-year-old female with osteoarthritis of the knee. In a further clinical study [18] and in a finite element analysis (FEA) [19], it was shown that the change in knee geometry typical for osteoarthritis and the loss of joint surface congruency may be an independent factor causing a medio-lateral translation of the knee joint and, consequently, an extrusion of the meniscus. Therefore, the decision was made to create a new model based upon a healthy knee joint. When considering the effects of changing the meniscus height on knee joint congruency, it was predicted that a change in the meniscus height may also be a cause for a medio-lateral translation of the knee joint. This could significantly affect the degree of extrusion. Thus, the goal was to assess the effect of a change in the meniscus height in both compartments on the medio-lateral shift of the knee joint and meniscus extrusion. Similarly, like in the previous paper, which was written by the same group of authors [16], it was considered that the Finite Element Method (FEM) should be the right tool to solve the above mentioned problem. In previous studies [20,21], the sensitivity of cartilage contact pressure on the size and shape of both menisci has been analysed. However, the effect of a change in the meniscus height on the lateral shift in a knee joint has not been analysed so far. Hence the subject has features of a scientific novelty.

## Materials and methods

### Geometry

**Fig 1:**
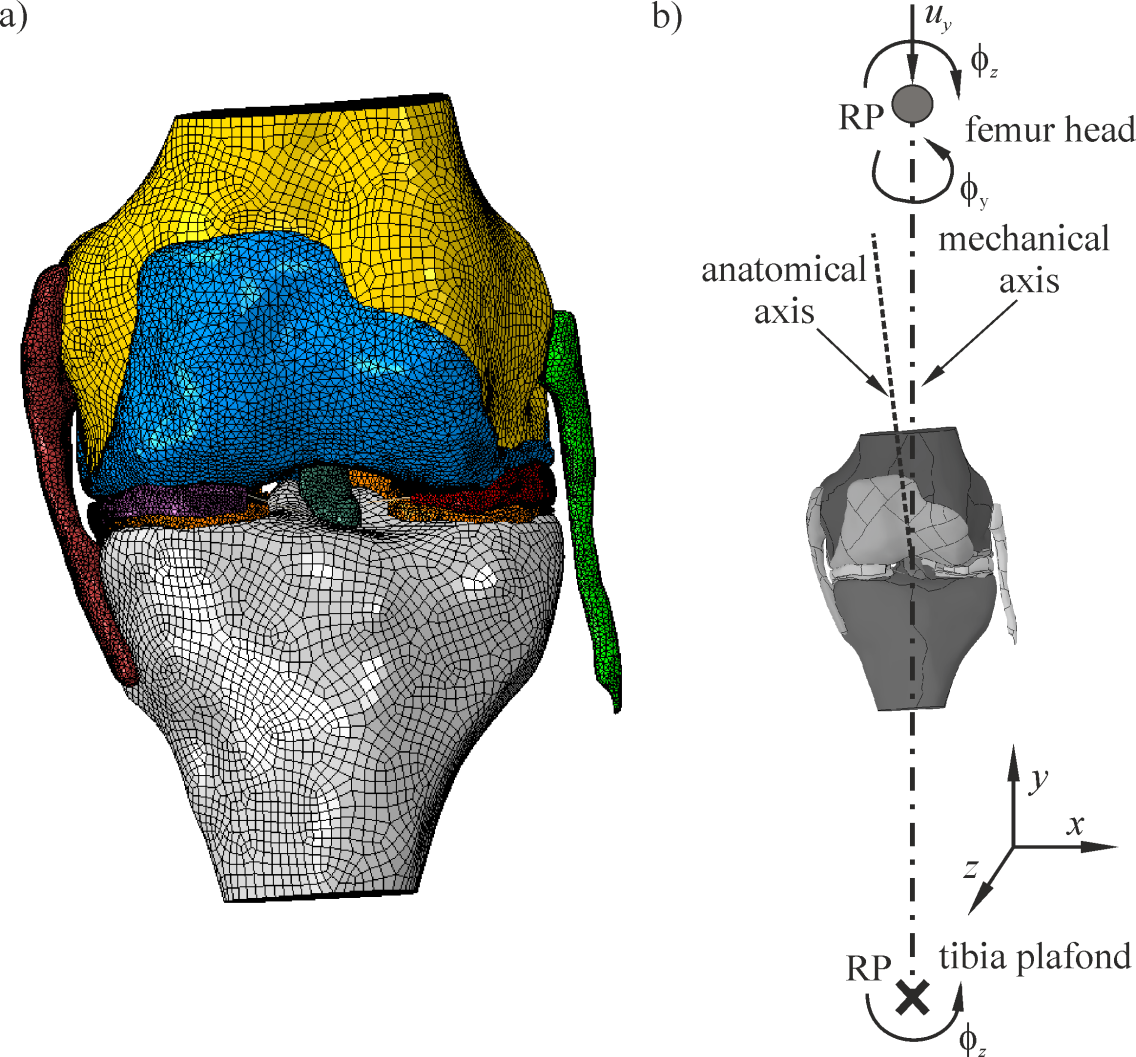
The knee joint model in a coronal view: a) geometry and the FEM discretisation, b) kinematic model, released degrees of freedom at the bone reference points (RPs).

The geometry of the knee joint was reconstructed based upon MRI scans of the left knee of a 43-year-old healthy female (height 1.75 m, weight 68 kg). The images were obtained in a full extension position by using a 1,5 T MRI scanner (Magnetom Aera, Siemens) in the axial, sagittal and coronal planes, with a resolution of 0.28125 mm. The spacing between the slices (images) was 0.7 mm in the axial plane and 3.6 mm in the sagittal and coronal planes. The study was approved by the Ethics Committee of the Medical University of Gdansk, Poland. The DICOM files were imported into Mimics Software (Materialise NV, Leuven, Belgium) which ensured a high accuracy of image segmentation. The segmentation of the upper airway by Mimics showed less than a 2% error in volumes when compared with the gold standard [22]. The similar methodology was used in the current study, so the accuracy of the knee joint segmentation should be of the same order. The comparison of MRI segmentation (pixel size 0.39 mm) with optical scans resulted in an absolute average error of 0.67 mm for the cartilage surfaces [23] and 0.56 mm for the bone surfaces [24]. The 3D geometry of the knee joint was created in semi-automatic segmentation that was performed by an experienced user. Based upon the 3D surface model, the FEM model was created in the commercial software, Abaqus 6.14 [25]. The knee joint model (see Fig 1A) consisted of bones (femur, tibia), menisci, articular cartilages and ligaments: anterior cruciate ligament (ACL), posterior cruciate ligament (PCL), medial collateral ligament (MCL) and lateral collateral ligament (LCL). This knee model, with the original geometry of the menisci, was used as the basic model for all of the other models.

The modified models were created by a uniform change of the cross-sectional height *h* of the lateral, or of the medial meniscus, in order to assess the influence of the meniscus shape on the knee joint biomechanics. The height of the lateral meniscus *h*_L_ was increased/decreased by 2.0 and 1.0 [mm], while the height of the medial meniscus *h*_M_ was modified by 1.8 and 0.9 [mm]. These values corresponded to 43.0% and 21.5% of the original average height, in the middle part of the lateral meniscus: *h*_L_ = 4.6 mm and the medial meniscus: *h*_M_ = 4.2 mm. The value of 43% was assumed as a standard deviation (SD) of the meniscus height, on the basis of experimental data for the middle part of meniscus allografts [26].

In the additional FEM models, denoted by CM-L, a numerical geometrical constraint that forced an equal medio-lateral translation of the tibia and the femur in the centre of the knee joint was introduced, (i.e. medio-lateral motion was possible, but the translation of the bones in the centre of the knee was the same). Consequently, for the CM-L models, the influence of the meniscus height on the meniscal external shift was solely investigated, because the relative bone motion was eliminated.

Summarising, in this paper, analyses were performed for the following models:

- model with the original geometry of the menisci (intact model);
- model with an increased height of the medial meniscus Δ*h*_M_ = +0.9 mm (model MMH↑);
- model with an increased height of the medial meniscus Δ*h*_M_ = +1.8 mm (model MMH↑↑);
- model with a decreased height of the medial meniscus Δ*h*_M_ = –0.9 mm (model MMH↓);
- model with a decreased height of the medial meniscus Δ*h*_M_ = –1.8 mm (model MMH↓↓);
- model with an increased height of the lateral meniscus Δ*h*_L_ = +1.0 mm (model LMH↑);
- model with an increased height of the lateral meniscus Δ*h*_L_ = +2.0 mm (model LMH↑↑);
- model with a decreased height of the lateral meniscus Δ*h*_L_ = –1.0 mm (model LMH↓);
- model with a decreased height of the lateral meniscus Δ*h*_L_ = –2.0 mm (model LMH↓↓);
- all of the above models with a constrained medio-lateral relative bone motion (models CM-L).

The kinematics of the femur and the tibia bones was defined by the so-called reference points (RPs). Due to disproportions in the stiffness between the bones and the cartilages, the first ones were defined as rigid structures [27]. The RPs were placed on the mechanical axis in the centre of the tibial plafond and in the centre of the femur head (Fig 1B). The distance between the RPs was calculated by using bone lengths that were estimated based upon the patient’s height and statistical data [28].

### Materials

The articular cartilages were assumed as being linear, elastic and isotropic, with an elastic modulus of *E* = 10 MPa and a Poisson ratio of *ν* = 0.45 [29,30]. The menisci were modelled as linear, elastic, and transversely isotropic, with the parameters as follows: *E*_*θ*_ = 120 MPa, *ν*_*rz*_ = 0.2 in a circumferential direction, *E*_*z*_ = *E*_*r*_ = 20 MPa, *ν*_*rθ*_ = *ν*_*zθ*_ = 0.3 in the axial and radial directions, and the shear moduli: *G*_*rz*_ = 8.33 MPa, *G*_*rθ*_ = *G*_*zθ*_ = 57.7 MPa [31,32].

The meniscal horn attachments were modelled as beams with a cross-sectional area corresponding to the area of the meniscal horns. The lengths of the meniscal attachments connecting the meniscus horns with the tibial plateau were estimated on the basis of the MRI images. The elastic moduli of the attachments were established as follows: 161 MPa for the lateral anterior, 96.3 MPa for the lateral posterior, 179 MPa for the medial anterior and 85.3 MPa for the medial posterior [33].

The behaviour of the ligaments was described by hyperelastic, nearly incompressible, transversely isotropic material, that was implemented by means of UMAT into Abaqus, with the strain-energy function:

$$\Phi =C_{1}({\bar{I}}_{1}-3)+\frac{1}{D_{1}}{\left(J-1\right)}^{2}+F_{2}(\lambda ),\;J=\det\;F$$ (1)

In the above equation ${\bar{I}}_{1}$ was the first invariant of the modified left Cauchy-Green tensor [34] and the constants *C*_1_ and *D*_1_ defined the matrix substance which was described by a nearly incompressible Neo-Hookean model [25,35]. The values of the volume ratio *J* computed at the end of analysis for the intact model were as follows: 0.99974 (ACL), 1.00001 (PCL), 0.99998 (MCL), 0.99958 (LCL). The function *F*_2_(*λ*) denoted the fibre family strain energy [36,37] and it fulfilled the conditions:

$$\lambda \frac{\partial F_{2}(\lambda )}{\partial \lambda }=\{\begin{array}{l} 0,\;\lambda \leq 1,\\ C_{3}\left(exp\left(C_{4}(\lambda -1)\right)-1\right),\;1<\lambda <\lambda^{*},\\ C_{5}\lambda +C_{6},\;\lambda \geq \lambda^{*}, \end{array}$$ (2)

where *λ* was the stretch of the fibres and the constants *C*_1_, *D*_1_, *C*_3_, *C*_4_, *C*_5_ are shown in Table 1. The characteristic value of the fibre stretch λ^*^ corresponded to the straightened fibres. The additional constant *C*_6_ was determined from the continuity condition of λ^*^.

**Table 1:** Ligament material parameters.

	*C*_1_ [MPa]	*D*_1_ [MPa^-1^]	*C*_3_ [MPa]	*C*_4_ [-]	*C*_5_ [MPa]	λ^*^ [-]	Source
ACL	1.95	0.01366	0.0139	116.22	535.039	1.046	[37]
LCL	1.44	0.00252	0.57	48.0	467.1	1.062	[38]
MCL	1.44	0.00252	0.57	48.0	467.1	1.062	[38]
PCL	3.25	0.0082	0.1196	87.178	431.063	1.035	[37]

The ligament attachments and the cartilage surfaces were connected to the bone surfaces by rigid constraints. The interactions between the specific articular surfaces and the menisci surfaces were introduced as a frictionless hard contact, with the use of an automatic stabilisation with a factor = 0.1, in order to improve the convergence of the analyses.

### Finite element mesh

The soft tissues were discretised by the 10-node solid elements (C3D10) with quadratic shape functions. The bones were meshed by using rigid shell triangular (R3D3) or quadrilateral (R3D4) elements. The meniscal horn attachments were modelled by using 3-node quadratic beam elements (B32). The average size of the elements was assumed to be about 1 mm. As a consequence, in the knee model with the intact geometry (see Fig 1A), the total number of nodes was 404,637, while the total number of finite elements was 265,257. The discretisation was verified by conducting convergence analyses. The double mesh refinement in the menisci and both cartilages, yielded an error smaller than 4%.

### Boundary conditions, initial state

All of the boundary conditions were applied to the bone RPs. When considering these constraints, five degrees of freedom (DOFs) remained fixed at the tibia RP, except for a varus/valgus rotation ϕ_*z*_ (Fig 1B). At the femur RP, a varus/valgus rotation ϕ_*z*_, a translation *u*_*y*_ in the direction of the mechanical axis, and an external/internal rotation ϕ_*y*_, were released (Fig 1B).

The initial state of the ligaments was imposed by introducing in each of them a total stretch equal to *λ*_0_ ≈ 1.05, yielding a deformation gradient of $F_{0}=diag[\lambda_{0},\lambda_{0}^{-\frac{1}{2}},\lambda_{0}^{-\frac{1}{2}}]$. The value of 5% was estimated based upon the literature [39]. In order to avoid a penetration of the contact surfaces in those models with an increased height of the meniscus, the femoral articular cartilage was translated in a top direction. Thus, in these models, the initial stretch was increased, in order to approximately assure the same resultant force in the ligaments, after the stretch phase, as in the knee model with the intact geometry.

The model of the knee joint was loaded by a compressive force of 1000 N. The force was placed in the femur RP and parallel to the mechanical axis direction. Static geometrical nonlinear analyses were carried out.

### Measured values

**Fig 2:**
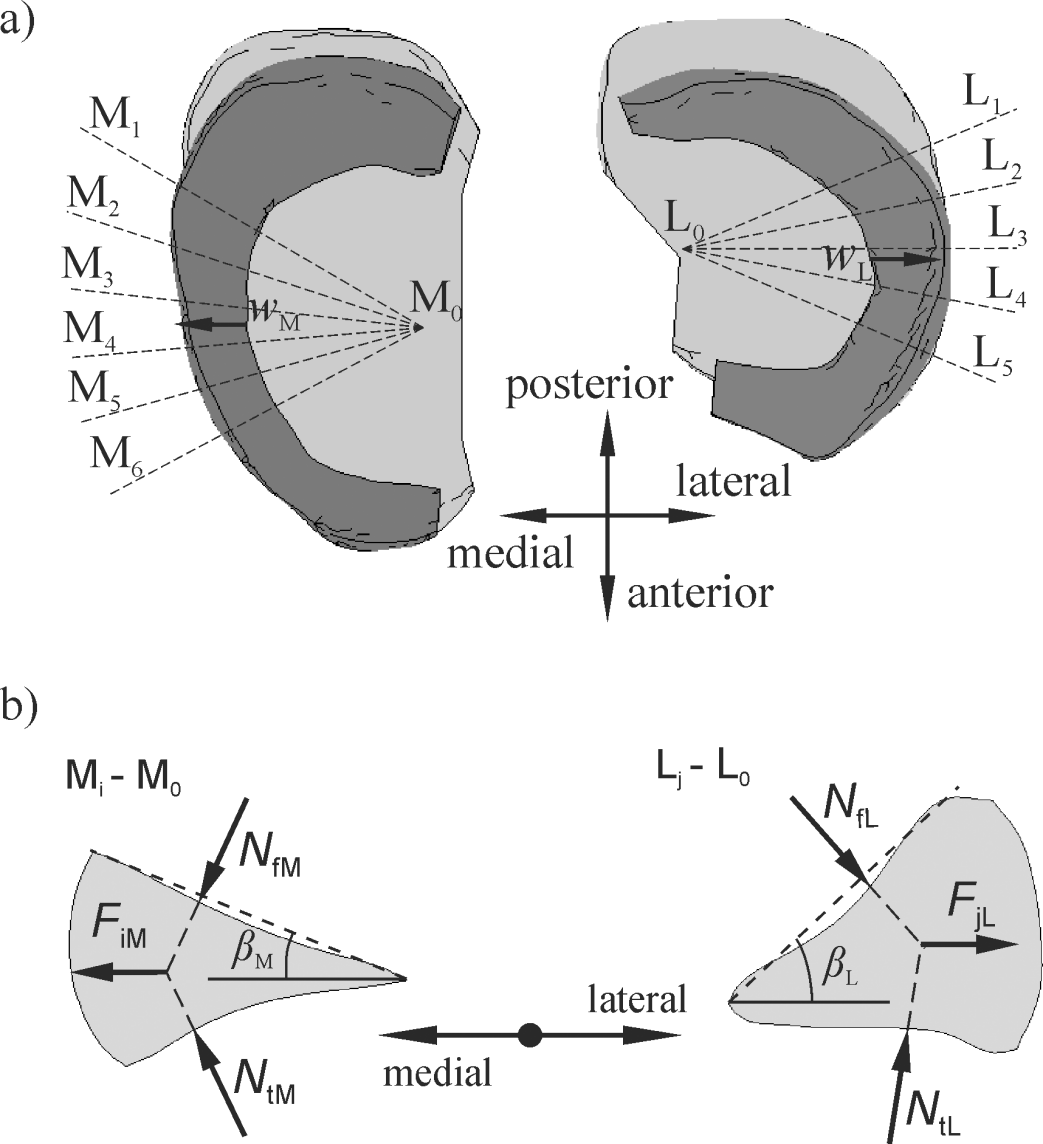
a) the definition of the meniscus external shifts *w*_M_ and *w*_L_ in an axial view and the positions of the M_i_-M_0_ and L_j_-L_0_ cross sections; b) the definition of the menisci angles *β*_M_ and *β*_L_ and the resultant forces *F*_iM_ and *F*_jL_ acting on the menisci in the M_i_-M_0_ and L_j_-L_0_ cross sections.

One of the main objectives of the study was to measure the meniscal external shifts and the relative bone translation while loading. The external shifts of the medial meniscus *w*_M_ and the lateral meniscus *w*_L_ were defined as the displacements of the centre point on the internal periphery of the meniscus (Fig 2A). The values of *w*_M_ and *w*_L_ were measured with respect to the tibia, in order to show the relative motion between the meniscus and the bone. Thus, the external shifts were equivalent to the differences in the meniscus extrusions between the loadings and the unloadings that have been previously measured in other papers [15,40]. The relative displacement *u* between the femur and the tibia was measured in a medio-lateral direction on the mechanical axis in the centre of the knee joint.

For the following discussion, let us define the two angles that are denoted respectively as: *β*_M_ and *β*_L_ (see Fig 2B). These angles were measured for the menisci at the M_i_-M_0_ and L_j_-L_0_ cross-sections, as shown in Fig 2A. In each model, the resultant values of the angles ${\bar{\beta }}_{M}$ and ${\bar{\beta }}_{L}$ were computed as the arithmetical averages of the angles *β*_M_ and *β*_L_, respectively. Fig 2B shows the concept of the resultant forces *F*_jL_ and *F*_iM_ acting on the menisci in each cross section of the lateral and the medial compartments, respectively. These forces arose due to the action of the contact forces *N*_f_ and *N*_t_ that were transmitted by the articular surfaces. In this paper, the extrusion forces *F*_L_ and *F*_M_ were defined as the medio-lateral components of the resultant contact forces for the menisci that were computed on the basis of *F*_jL_ and *F*_iM_, from all of the meniscus cross sections. In the models denoted by CM-L, the constraint of the medio-lateral relative bone motion was associated with the medio-lateral constraint force *F*_M-L_.

As stated in the introduction the goal of this study was to assess the effects of a change in the meniscus height in both of the compartments on the medio-lateral shift of the knee joint and meniscus extrusion. These effects can be effectively assessed by measuring the congruence between the contact surfaces in the knee. A study of the literature has shown that different definitions of knee joint congruence and its evaluation exist [41]. Here, the congruence measurement *CM*, as proposed in [41], was applied, in order to quantify the joint congruency. Another conception has been proposed for the MRI images, see for instance [42]. In this study *CM* was computed separately for both knee compartments as

$$CM=\frac{F}{p_{0}}$$ (3)

where *F* was the resultant contact force and *p*_0_ was the peak contact pressure in a given compartment. The total contact areas in the medial compartment and the lateral compartment were used as additional global measurements of congruence. The total contact force and the contact area, were computed by Abaqus by using the variables: CFNM and CAREA, respectively for a given pair of contact surfaces.

## Results

**Fig 3:**
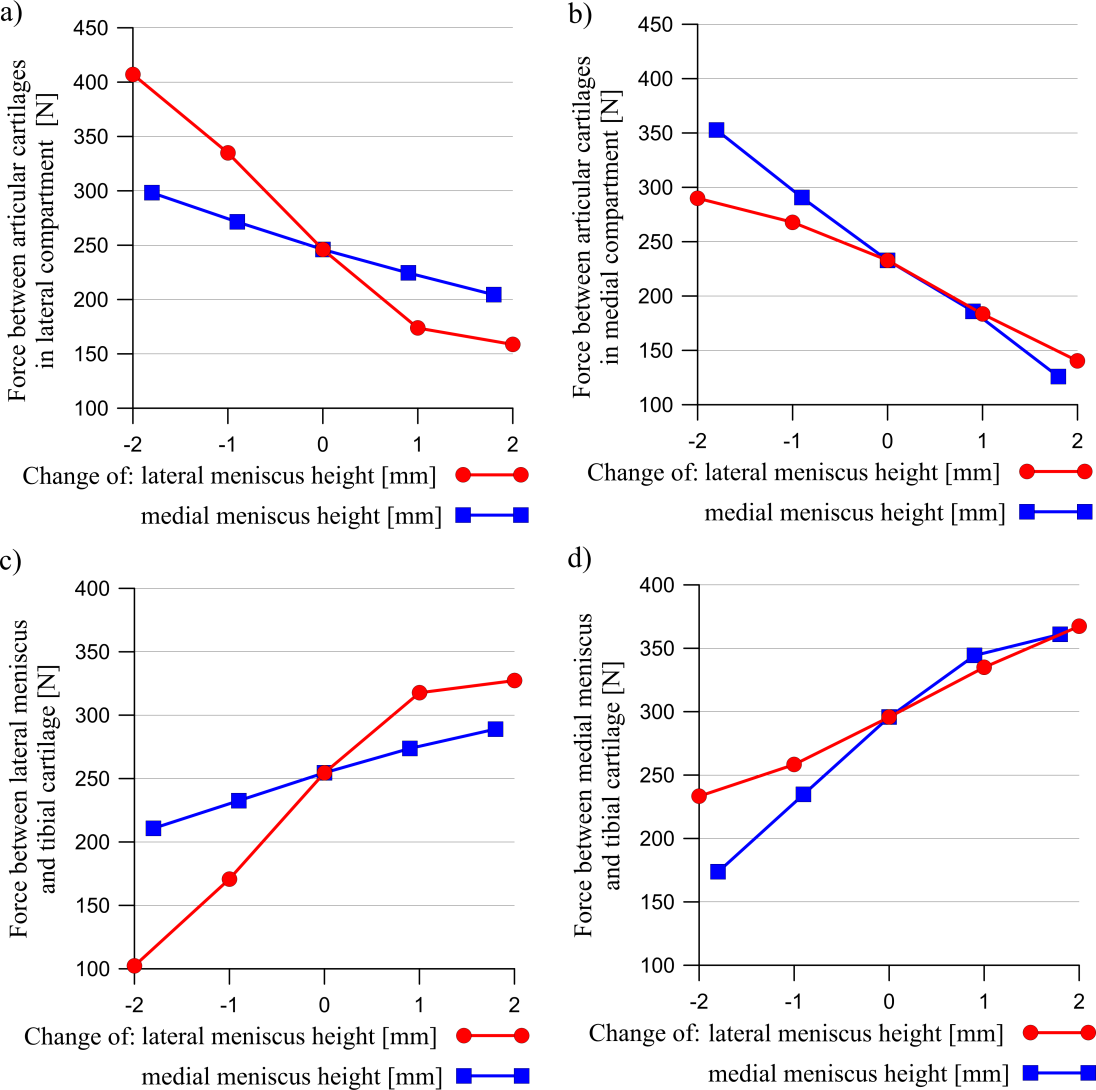
The influence of a change in the lateral meniscus height (red line) or the medial meniscus height (blue line) on the resultant contact forces between the articular cartilages in: a) the lateral compartment, b) the medial compartment and the resultant contact forces between the meniscus and the tibial cartilage in: c) the lateral compartment, d) the medial compartment.

**Fig 4:**
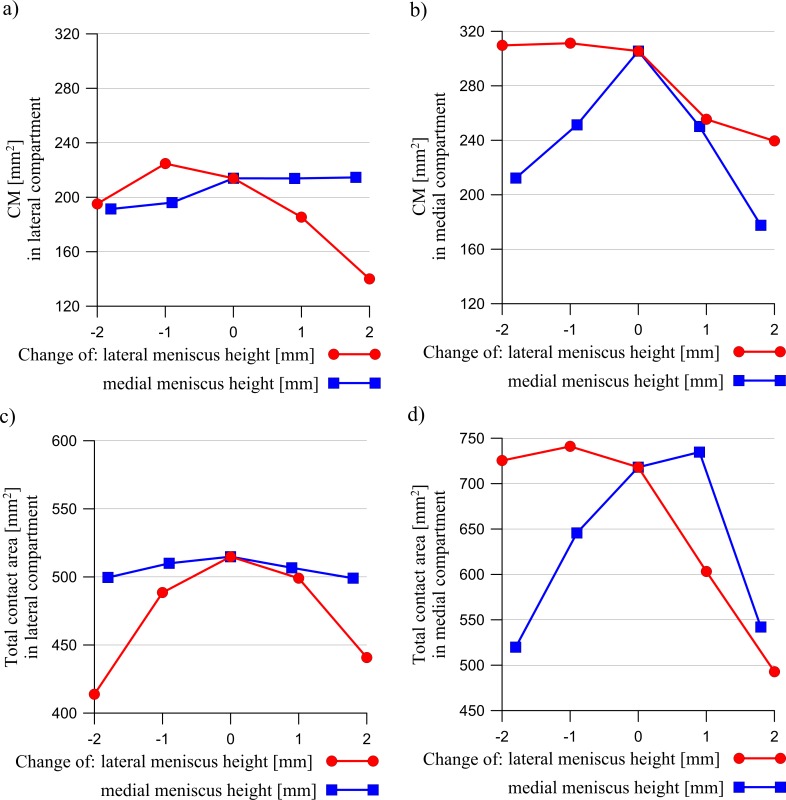
The influence of a change in the lateral meniscus height (red line) or in the medial meniscus height (blue line) on the congruence measurement *CM* in: a) the lateral compartment, b) the medial compartment and the total contact area in: c) the lateral compartment, d) the medial compartment.

**Fig 5:**
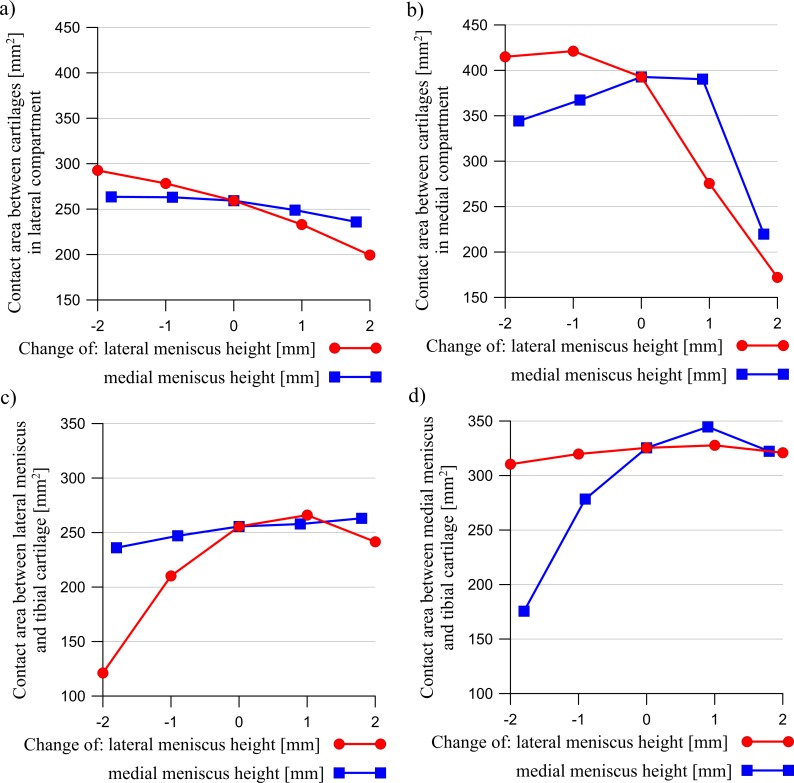
The influence of a change in the lateral meniscus height (red line) or in the medial meniscus height (blue line) on the contact areas between the articular cartilages in: a) the lateral compartment, b) the medial compartment and the contact areas between the menisci and the tibial cartilage in: c) the lateral compartment, d) the medial compartment.

The results presented below were obtained for a load of 1000 N. A change in the meniscus height meaningfully affected the distribution of the contact forces acting between the articular cartilages and between the cartilages and the menisci (Fig 3). The curves in Fig 3 show that an increase in the meniscus height in one compartment caused a drop in the value of the force transmitted between the cartilages, with an increase of force between the cartilage and the meniscus in both compartments. However, the changes in the modified compartment were greater. A modification of the meniscus cross sectional shape generally decreased the congruency of the knee joint (see Fig 4). The congruence measure *CM* and the total contact area have shown that the knee joint congruency was mainly affected in the modified compartment. A slight increase of *CM* in both compartments was observed only in the case of a small reduction in the lateral meniscus height. The redistribution of the contact forces and the reduced congruency of the knee joint caused changes in the contact areas (see Fig 5). An increase in the meniscus height caused a drop in the value of the contact area between the articular cartilages, while a decrease in the meniscus height in one compartment, caused a large decrease in the contact area between the meniscus and the cartilage in this compartment.

**Fig 6:**
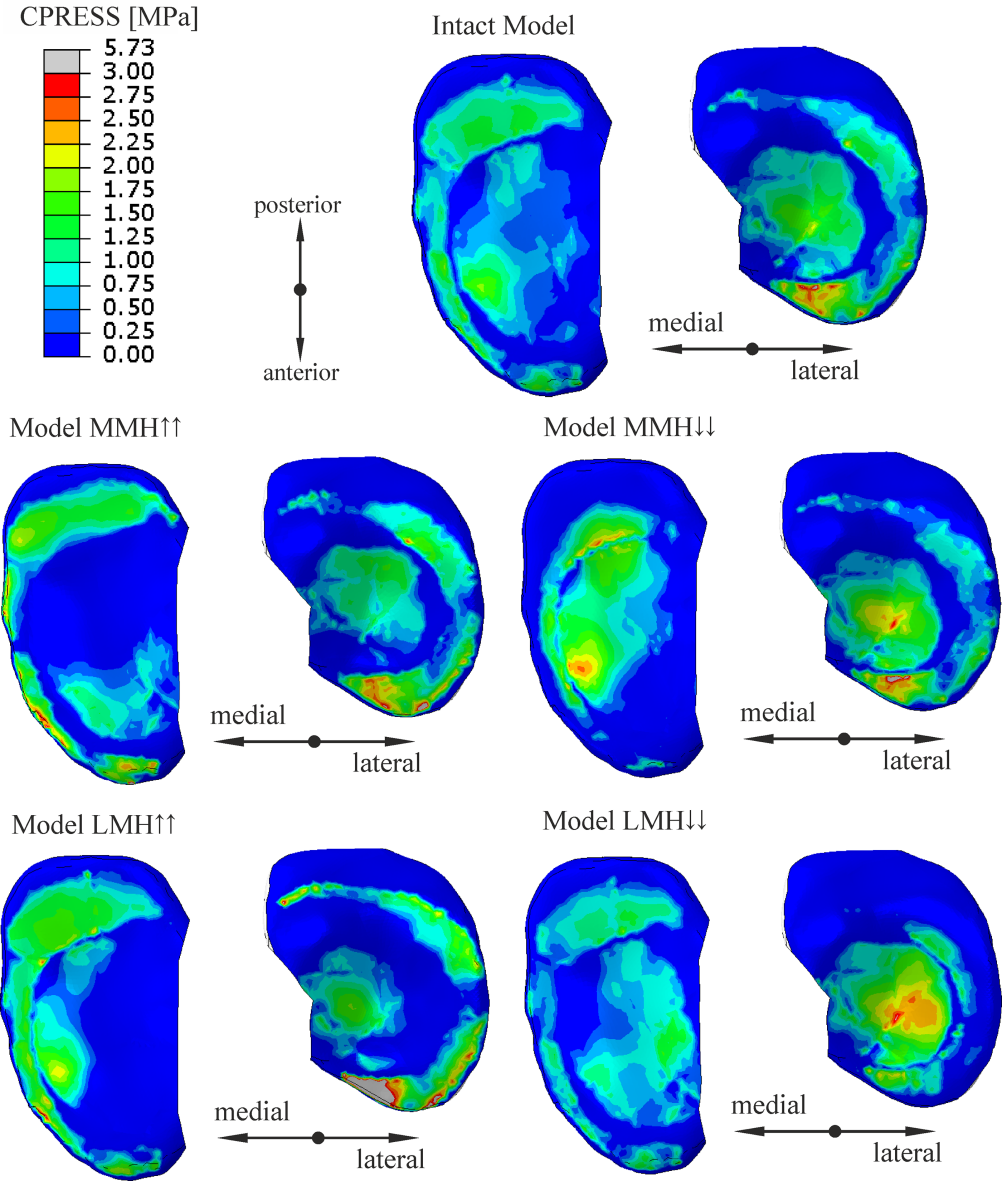
A comparison of the contours of the contact pressures on the tibial articular cartilages for the different knee models: Intact model with the original geometry of the menisci; model MMH↑↑ with an increased height of the medial meniscus; model MMH↓↓ with a decreased height of the medial meniscus; model LMH↑↑ with an increased height of the lateral meniscus; model LMH↓↓ with a decreased height of the lateral meniscus; Δ*h*_M_ = ±1.8 mm, Δ*h*_L_ = ±2.0 mm.

The contours of the contact pressures (Fig 6) show that a change in the meniscus’s geometry caused a redistribution of the stresses in the modified compartment. A maximal value of contact pressure was observed under the anterior horn of the lateral meniscus in the model with an increased height of the lateral meniscus (model LMH↑↑).

**Fig 7:**
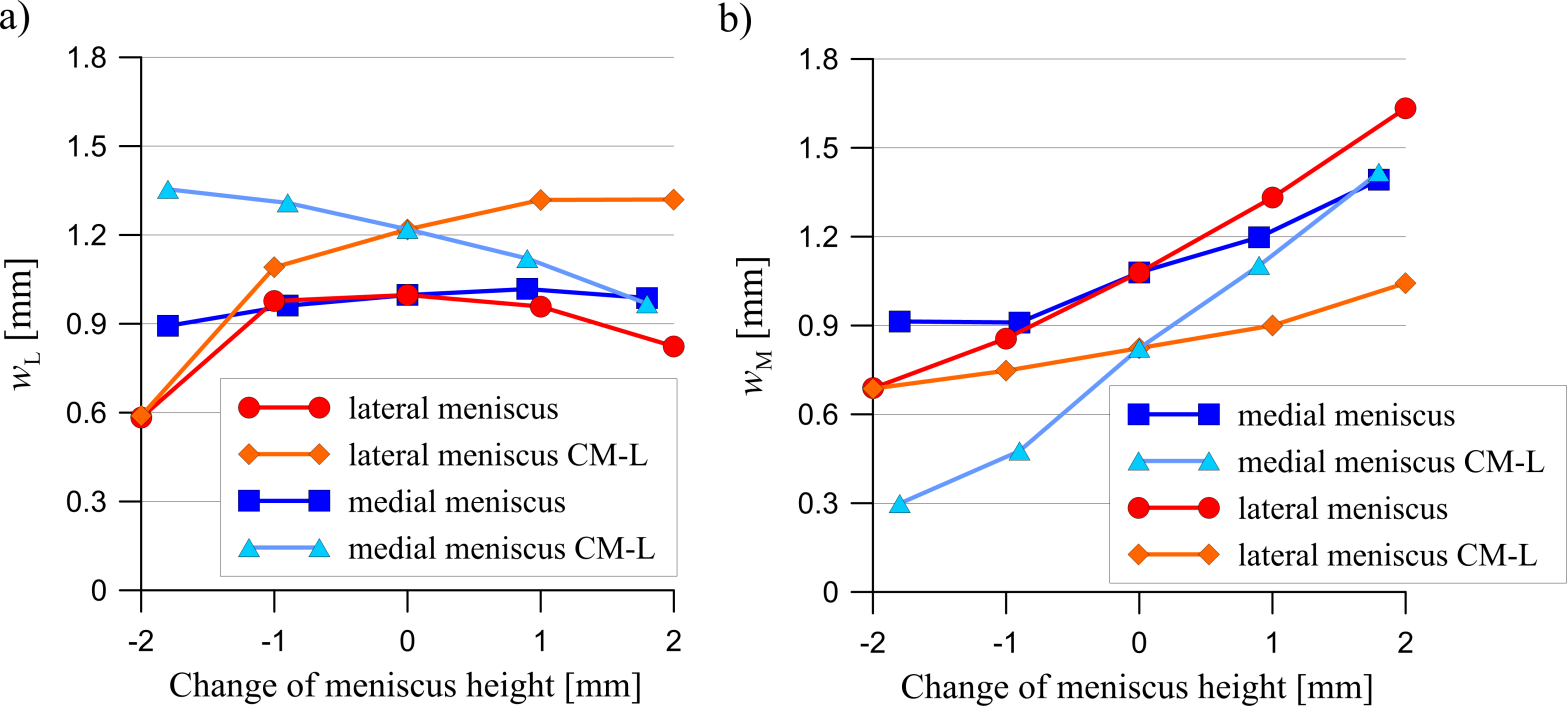
The influence of a change in the lateral meniscus height (red line) or in the medial meniscus height (blue line) on: a) the external shift of the lateral meniscus *w*_L_; and b) the external shift of the medial meniscus *w*_M_. CM-L–models with a constrained medio-lateral relative bone motion.

A change in the meniscus height Δ*h* caused an alteration of the average menisci angles ${\bar{\beta }}_{M}$ and ${\bar{\beta }}_{L}$, as presented for each model in Table 2. In the models CM-L, the medio-lateral relative bone motion was constrained, thus *u* = 0. This constraint was associated with the medio-lateral constraint force *F*_M-L_ (see Table 2). The values of *u* were computed for the models with a free relative bone motion, for which *F*_M-L_ = 0. A maximal value of *u* was observed in the model with an increased height of the lateral meniscus (Δ*h =* +2.0 mm) and there was a maximal value of *F*_M-L_ in the model with a decreased height of the medial meniscus (Δ*h =* –1.8 mm). Fig 7 shows that the dependency between the meniscus height and the meniscal external shift was different for the lateral and the medial meniscus. An increase in the meniscus height resulted in a greater external shift of the medial meniscus (Fig 7B). While in the case of a lateral meniscus (Fig 7A), a similar relationship was only observed in those models with a change in height of the lateral meniscus and with a constrained medio-lateral relative bone motion (CM-L).

**Table 2:** A comparison of the average menisci angles ${\bar{\beta }}_{M}$ and ${\bar{\beta }}_{L}$, forces FM-L and relative medio-lateral bone translation u. The medio-lateral constraint forces *F*_M-L_ were calculated for the models with a constrained medio-lateral relative bone motion. A positive value of *u* denoted the medial relative translation of the femur with respect to the tibia. Intact model with the original geometry of the menisci; models MMH↑, MMH↑↑ with an increased height of the medial meniscus; models MMH↓, MMH↓↓ with a decreased height of the medial meniscus; models LMH↑, LMH↑↑ with an increased height of the lateral meniscus; models LMH↓, LMH↓↓ with a decreased height of the lateral meniscus.

	${\bar{\beta }}_{L}$ [°]	${\bar{\beta }}_{M}$ [°]	${\bar{\beta }}_{L}$ – ${\bar{\beta }}_{M}$ [°]	*F*_M-L_ [N]	*u* [mm]
Model MMH↓↓	32.7	14.5	18.2	158.3	0.899
Model MMH↓	32.7	19.3	13.4	124.7	0.650
Intact model	32.7	23.9	8.8	79.6	0.392
Model MMH↑	32.7	28.2	4.5	33.4	0.159
Model MMH↑↑	32.7	32.1	0.6	-7.8	-0.060
Model LMH↓↓	21.8	23.9	-2.1	1.2	-0.008
Model LMH↓	27.9	23.9	4.0	33.7	0.155
Intact model	32.7	23.9	8.8	79.6	0.392
Model LMH↑	37.4	23.9	13.5	130.2	0.752
Model LMH↑↑	40.8	23.9	16.9	132.6	1.484

**Fig 8:**
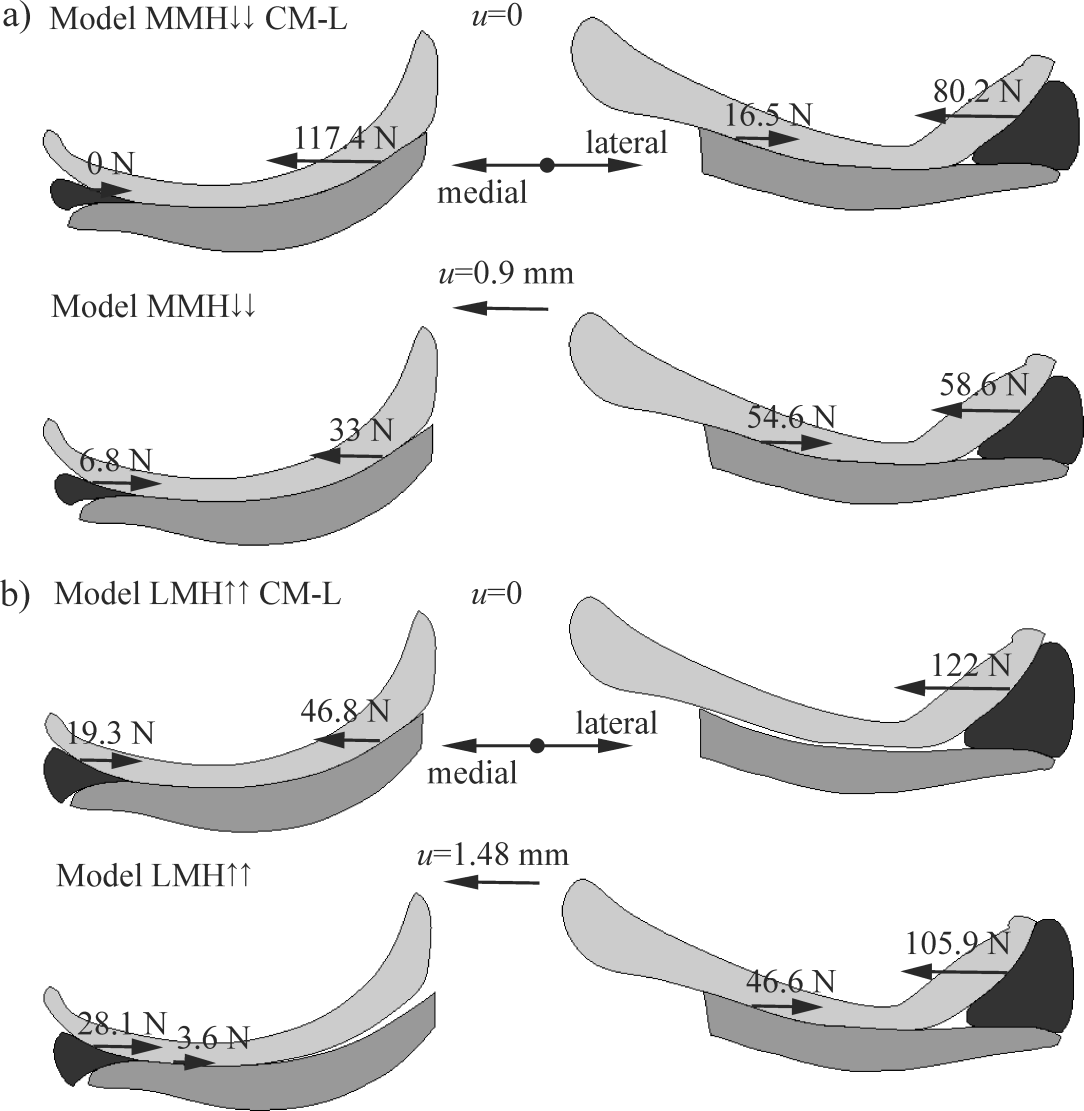
The influence of the relative bone motion *u* on the medio-lateral components of the contact forces acting on the femur condyle in: a) model MMH↓↓ with a decreased height of the medial meniscus; and b) model LMH↑↑ with an increased height of the lateral meniscus; CM-L–models with a constrained medio-lateral relative bone motion.

**Fig 9:**
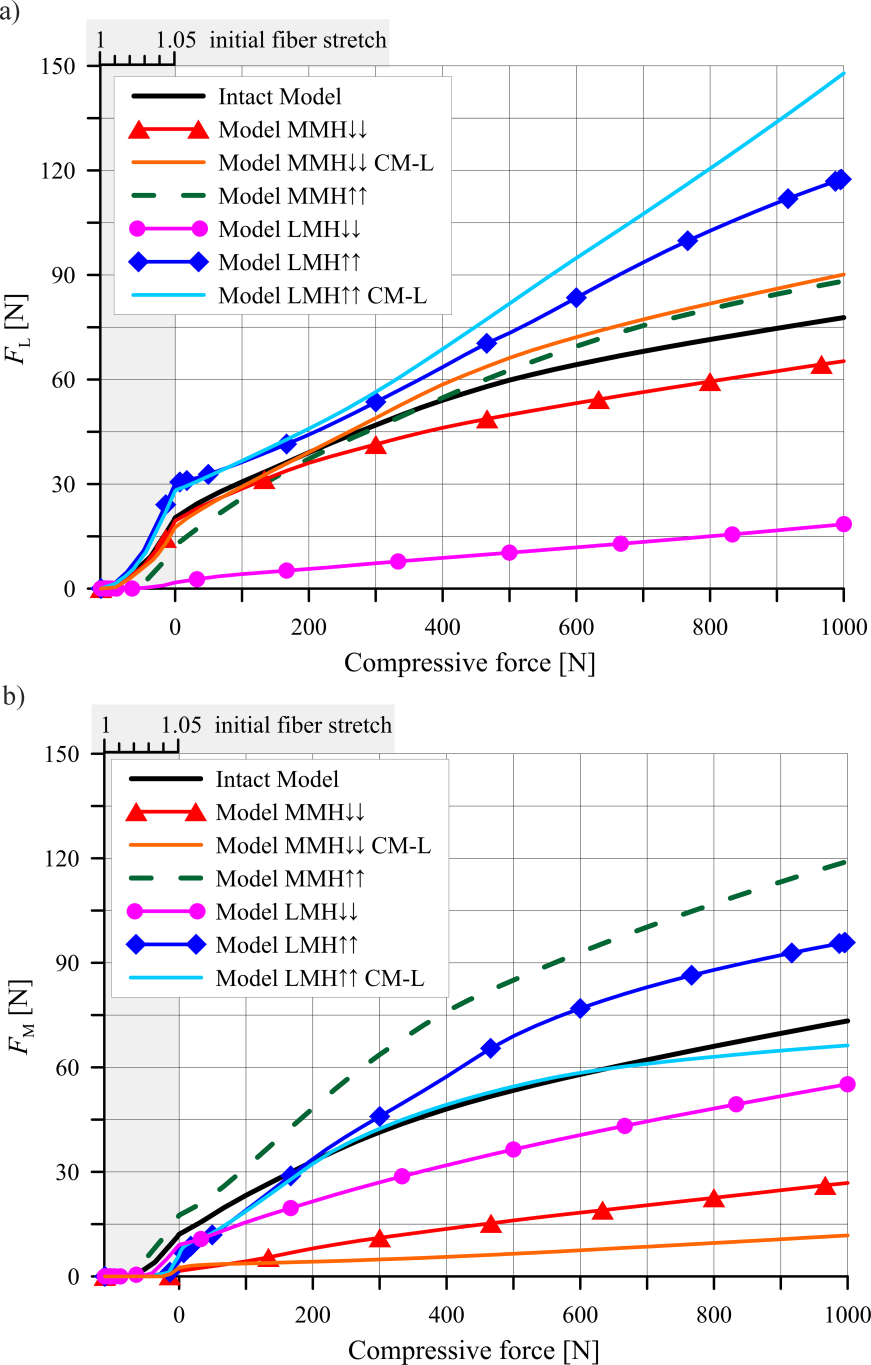
The extrusion forces: a) *F*_L_ acting on the lateral meniscus; and b) *F*_M_ on the medial meniscus, in a function of the compressive force. Intact model with the original geometry of the menisci; models MMH↑, MMH↑↑ with an increased height of the medial meniscus; models MMH↓, MMH↓↓ with a decreased height of the medial meniscus; models LMH↑, LMH↑↑ with an increased height of the lateral meniscus; models LMH↓, LMH↓↓ with a decreased height of the lateral meniscus; CM-L–models with a constrained medio-lateral relative bone motion.

Fig 8 shows how the relative bone motion changed the medio-lateral components of the resultant contact forces that acted on the femoral cartilages. The forces were presented only in the models with the greatest value of *u*, namely: model MMH↓↓ and model LMH↑↑. The relative translation *u* resulted in an increment of the forces acting between the articular cartilages in the lateral compartment and a decrement in the medial compartment. Additionally, the relative translation *u* decreased the differences in the forces acting on the menisci in the lateral and medial compartments. The dependence of the extrusion forces, as functions of the compressive load acting on the knee is presented in Fig 9. The greatest values of the extrusion forces *F*_M_ and *F*_L_ were observed for those models with an increased height of the meniscus.

## Discussion

Firstly, the results of the basic knee model with intact geometry were confirmed to be consistent with the literature data [43,44,45,46], (see comparison of the values of the maximal contact pressure, the average contact pressure and the total contact area in Table 3). Since this paper was not clinical in nature, it was decided that a literature-based validation would be sufficient [47].

**Table 3:** Literature-based comparison of the values of the total contact area, the maximal and the average contact pressure for the basic knee model with intact geometry.

	Total contact area [mm^2^]	Maximal contact pressure [MPa]	Average contact pressure [MPa]
800 N			
Present study	1160.99	2.62	0.72
[43]	900	2.5	0.86
1000N			
Present study	1232.63	2.95	0.83
[44]	1083	2.96	1.0
[45]	1044.54	3.09	-
[46]	1300 ± 300	-	0.8 ± 0.2

Next, the heights of the meniscus in the medial compartment (models MMH↓, MMH↓↓) and in the lateral compartment (models LMH↓, LMH↓↓) were reduced. The values of the meniscal external shift in the models with a decreased height of the meniscus (Fig 7) confirmed a previous hypothesis [16,48] that a flattened meniscus has a smaller tendency for an external shift due to a decreased value of the extrusion force (Fig 9). A decrease in the force acting between the flattened meniscus and the cartilage and increase in the force acting between the cartilages (Fig 3) were observed in these models. The observations that a reduction in the meniscus height decreased the contact area between the cartilage and the meniscus (Fig 5) are consistent with the reported work of [20].

The models with an increased meniscus height in the medial compartment (models MMH↑, MMH↑↑) and in the lateral compartment (models LMH↑, LMH↑↑) were then investigated. In these models, an increase in the force acting between the modified meniscus and the cartilage (Fig 3) was not associated with an increase in the contact area (Fig 5), due to the deteriorated congruency of the contact surfaces (Fig 4). In addition, the authors’ hypothesis was confirmed, an increase in the meniscus height increased the extrusion force (Fig 9) and that may have caused an excessive external shift of the meniscus (Fig 7) [48].

In the model LMH↓↓, with a flattened lateral meniscus, a decrease in the external shift of the lateral meniscus *w*_L_ was observed, as well as in the medial meniscus *w*_M_, with an unchanged geometry (Fig 7). The value of *w*_M_ in model LMH↓↓ decreased more than it did in model MMH↓↓, with a flattened medial meniscus. Furthermore, an increase in the lateral meniscus height resulted in the greatest increase of *w*_M_ and a slight decrease of *w*_L_. Hence, the authors’ hypothesis that a change in the geometry of the medial meniscus and the lateral meniscus might affect extrusion in a different way was confirmed. These phenomena were caused by a change in the medial translation of the femur relative to the tibia (see the values of *u* in Table 2), which was confirmed by the values of *w*_M_ and *w*_L_ in the models CM-L, with a constrained medio-lateral relative bone motion (see Fig 7). A comparison of the values of the external shifts, between the models with free and constrained medio-lateral relative bone motion, allowed for a conclusion that the medio-lateral knee joint translation could be treated as an overriding risk factor for a meniscal extrusion process (see Fig 7). Patel et al. investigated the impact of a knee joint loading on a meniscal extrusion in normal individuals [15]. They found in MRI study that only the medial meniscus extrusions were significantly increased during the loading. It was shown here that a relative medio-lateral bone motion may be the reason for a low meniscal external shift in the lateral compartment.

The medio-lateral knee joint translation was dependent on the geometry of the articular surfaces and the ligament integrities [49,50]. Belvedere et al. carried out femur and tibia tracking in cadaver knees during joint motion [51]. They reported for the first time that the relative femoral-to-tibial medio-lateral translation which occurs in a normal knee joint can be as large as 35% of the tibial plateau width during knee flexion. In this current research, the changes of geometry in the medial meniscus influenced the medio-lateral relative bone motion to a smaller extent, contrary to the changes in the lateral meniscus (Table 2). The possible explanation is that in the lateral compartment, where the articulating femorotibial cartilage surfaces are both convex, the congruency is more dependent on the cross sectional shape of the meniscus, than it is in the medial compartment, where the congruency is dependent on the matching articular surfaces. In the present work, the authors’ hypothesis was confirmed, relative medio-lateral translation of the bones may be dependent on the geometry of the menisci.

A comparison of the resultant contact forces in the models with free and constrained medio-lateral relative bone motion has helped us to understand the mechanisms of medio-lateral motion. It was observed that the main reason for a medio-lateral relative bone translation was the difference between the values of ${\bar{\beta }}_{L}$ and ${\bar{\beta }}_{M}$ (Fig 2B, Table 2). The consequences of a greater angle of ${\bar{\beta }}_{L}$ in the lateral compartment will result in greater contact forces acting on the femur in a medial direction and an instability of the knee joint in an original position for *u* = 0 (Fig 8). A measurement of this instability was a constraint reaction *F*_M-L_ between the femur and the tibia in the models with a constrained medio-lateral relative bone motion (Table 2). The force *F*_M-L_ was approximately proportional to the difference between the angles of ${\bar{\beta }}_{L}$ and ${\bar{\beta }}_{M}$. A relationship between translation *u* and *F*_M-L_ (Table 2) was also observed. The relative bone motion assured a balance of the forces acting between the bones in a medio-lateral direction. Thus, the medial translation of the femur relative to the tibia reduced the contact forces acting in a medial direction and increased the contact forces counteracting this motion (Fig 8). A change in the medio-lateral resultant contact forces was meaningfully greater between the articular cartilages than between the menisci and the femoral condyle (Fig 8). Therefore, the geometry of the articular surfaces may also have a big influence on the relative bone motion. The medial translation of the femur relative to the tibia increased the extrusion force *F*_M_, and thereby, the meniscal external shift *w*_M_ in the medial compartment. Simultaneously, it reduced the values of *w*_L_ and *F*_L_ in the lateral compartment (see Fig 7 and Fig 9). Hence in the model with an increased height of the lateral meniscus, the medial meniscus was strongly loaded in its centre part and it was pushed out of the knee joint.

The presented results are subject to several limitations. The knee model geometry lacked capsule and meniscal ligaments which may have had some influence on the presented findings. The linear, elastic isotropic material model was used for the articular cartilages and the transversely isotropic model was used for the other soft tissues. This was because only the initial time-independent analyses were performed. Since the influence of articular cartilage stiffness on meniscus biomechanics is low, the assumed simplified model of the cartilage has not considerably affected the results [52,53]. The load was applied as a static one, without any dynamical effects. Therefore, the stress values due to a dynamical overload were not captured. The potential segmentation error may affect the absolute values that have been reported in this paper. However, the conclusions that were based on a comparison of the results between the specific models will not change, because errors during the segmentation process were systematic errors present in all of the models. Analyses were performed on only one patient-specific FE model so it is difficult to generalise the results and further investigations will have to be performed on the subject.

The most relevant conclusion was that the external shift of the meniscus depended on both its geometry and its femoral-to-tibial medio-lateral translation, which could be treated as an overriding risk factor for a meniscal extrusion process. Thus, changing the position of the meniscus can be regarded not only as a symptom of the damage in the structure of the meniscus (hoop tension), but also as an instability of the knee joint. The conclusions drawn from this work can help in the interpretation of the radiological features of a meniscus extrusion, especially in the absence of any meniscal damage. It may also help in the selection of optimal meniscal allografts.
